# A novel stable biomimetic adhesive coating for functionalization of orthodontic brackets against bacterial colonization and white spot lesions

**DOI:** 10.1186/s12903-024-05313-3

**Published:** 2025-01-04

**Authors:** Lamia Singer, Sabina Karačić, Gabriele Bierbaum, Brianne Palmer, Christian Kirschneck, Christoph Bourauel

**Affiliations:** 1 Oral Technology, University Hospital Bonn, 53111 Bonn, Germany; 2 Department of Orthodontics, University Hospital Bonn, 53111 Bonn, Germany; 3 Institute of Medical Microbiology, Immunology and Parasitology, University Hospital Bonn, 53127 Bonn, Germany; 4 Bonn Institute of Organismic Biology, Division of Palaeontology, University of Bonn, 53115 Bonn, Germany

**Keywords:** Orthodontic brackets, Polydopamine, Biofilm formation, Surface bioactivity, pH change

## Abstract

**Background:**

This study aimed to evaluate the efficacy of polydopamine (PDA) functionalization on orthodontic brackets in inhibiting biofilm formation and promoting surface bioactivity to buffer the acidity of caries-causing bacteria around orthodontic brackets and prevent demineralization. The stability of the coating in artificial saliva (AS) and distilled water was evaluated, along with its effect on pH changes in simulated body fluid (SBF) and distilled water.

**Methods:**

Maxillary incisor orthodontic brackets underwent PDA functionalization using a dopamine hydrochloride solution following a specific protocol. Biofilm formation on both control (Br-0) and coated (Br-PDA) brackets was assessed immediately after coating and after two months of aging (Aged Br-PDA) in artificial saliva. The adherent biofilm bacteria on brackets were quantified with colony count assessment and optical density. Surface morphology, Bioactivity, and coating stability were analyzed using Scanning Electron Microscopy (SEM). Coated and uncoated samples were immersed in SBF and deionized water, and pH changes were monitored over 7 days using a pH meter.

**Results:**

PDA-functionalized brackets, both freshly coated (1.08 OD) and aged for two months (1.6 OD), showed significantly reduced biofilm formation compared to non-functionalized control brackets (2.07 OD), with p-value < 0.05. This reduction was confirmed through optical density measurements and colony-forming unit (CFU) counts (1.63E + 06, 4.53E + 07, and 7.56E + 07 respectively, p-value < 0.05). SEM analysis revealed alterations in surface morphology and composition, suggesting enhanced biointeraction in the coated brackets. Stability assessments in artificial saliva and deionized water demonstrated the durability of the coating. pH measurements indicated minimal changes in SBF and water, with PDA-functionalized brackets showing slight alterations.

**Conclusions:**

Our research findings suggest that PDA-functionalized brackets possess promising antimicrobial properties and stability, offering potential applications in orthodontic treatment to mitigate biofilm formation and prevent white spot lesions around orthodontic brackets. Further investigation is required to optimize the coating formulation and explore its long-term efficacy in clinical settings.

## Introduction

Orthodontic treatment often involves brackets bonded to teeth to align the dentition properly and enhance oral function and aesthetics [1]. However, these brackets can become sites for plaque accumulation and retention of bacteria near brackets and elastic bands leading to the demineralization of the adjacent tooth enamel and subsequent dental decay [2]. The presence of orthodontic brackets and bands on the tooth surfaces presents difficulties for patients in maintaining proper brushing and oral hygiene routines. Consequently, this raises the likelihood of developing cavities [3].

*Streptococcus mutans* (*S. mutans*), a common cariogenic bacterium, is pivotal in biofilm development [4]. It rapidly adheres to tooth surfaces, secreting acids and generating extracellular polysaccharides, facilitating biofilm formation [5]. These biofilms create a protective milieu for acid-producing bacteria, enhance the retention of harmful substances, and contribute to the gradual demineralization of tooth enamel [6]. This demineralization often manifests as unsightly white spot lesions (WSL) [7]. Notably, WSL can become visible around brackets within a month of bonding, with reported prevalence rates of 38% at 6 months 46% at 12 month, and 50% by the end f the orthodontic treatment [8].

To address this issue, considerable research has been dedicated to developing innovative approaches aimed at preventing bacterial adhesion and enhancing the brackets’ biocompatibility [9, 10]. Some studies have shown that coating brackets with functional materials, such as chitosan, and nano-coating materials like silver, silver-platinum, titanium, zinc oxide, and copper oxide have exhibited an adequate antibacterial effect by decreasing the adherence of *Streptococcus mutans* on the orthodontic brackets [11, 12].

It has been demonstrated that adhesives in marine mussels are composed of a catechol amino acid known as 3,4-dihydroxyphenylalanine (DOPA). Combined with the amino acid lysine, this compound facilitates adhesion to various surfaces, even in moist and saline environments [13]. Drawing inspiration from these properties, a new coating material, polydopamine (PDA), has been engineered from dopamine, which is a catechol derivative [14]. PDA exhibits remarkable adherence capabilities to nearly all substrates, mirroring the versatile anchoring abilities observed in mussel-derived adhesives [15].

Polydopamine (PDA) has recently been used as a versatile functionalization tool that could be applied to a range of substrates to improve wettability, and biocompatibility and to allow for further bio-functionalization [16]. One of the main advantages of PDA is its easy application, which is achievable under slightly basic conditions (around pH 8.5) via the oxidative polymerization of dopamine. This process results in the formation of a biocompatible layer that firmly adheres to the surface of various materials [17]. PDA’s ability to adhere to diverse substrates and its biocompatibility makes it an attractive candidate for enhancing the bioactivity of materials used in medical implants, drug delivery systems, and biosensors. Additionally, the facile and environmentally friendly synthesis of PDA further adds to its appeal in various fields [17].

Recently, PDA has been used as a candidate to induce the deposition of calcium phosphate minerals on various substrate materials as a biomimetic coating [18]. According to the findings, surfaces treated with polydopamine coating and anchored catecholamine moieties facilitate the enrichment of calcium ions at the interface through binding with Ca^2+^ [18]. This enrichment promotes the formation of hydroxyapatite (HA) crystals aligned with the c-axes, parallel to the polydopamine layer [19].

Furthermore, PDA demonstrates promising attributes in antimicrobial applications [20]. Its adhesive properties enable the creation of coatings that repel pathogenic organisms and hinder microbial adhesion on surfaces. PDA possesses outstanding photothermal conversion ability, abundant catechols, and secondary amine structures [21]. The reactive oxygen species (ROS) and hydrogen peroxide produced by PDA can oxidize the sulfur-containing amino acids of cellular and membrane proteins and DNA thus positioning PDA as a pioneering and efficient biological antibacterial agent [22, 23]. The diverse abilities of PDA continue to drive research efforts for new applications, showing its significant influence in different scientific fields.

A novel approach is emerging in the development of drug-free, biomimetic, and efficient methods to prevent biofilm formation on dental appliances. Coating orthodontic brackets with functional and safe materials offers significant potential in addressing the challenges of demineralization. Therefore, the motivation behind this study was to explore the use of polydopamine (PDA), a biomimetic compound known for its antimicrobial and remineralization properties, as a coating material. The study aimed to apply PDA to orthodontic brackets to reduce the occurrence of white spot lesions by facilitating the deposition of calcium and phosphate at the bracket-tooth interface, buffering acidic conditions, and providing anti-biofilm effects. The null hypothesis is that polydopamine-coated brackets will not significantly reduce biofilm formation and that the coating will not maintain stability over time.

## Materials and methods

### Materials

Maxillary incisor orthodontic brackets (Discovery Smart, Dentaurum, Ispringen, Germany), dopamine hydrochloride (Sigma Aldrich Co, St. Louis, MO, USA), TRIS, different reagents for simulated body fluid and artificial saliva preparation (Sigma Aldrich Co, St. Louis, MO, USA), *S. mutans* (DSMZ 20523) were used in the study.

### Coating procedure and sample preparation

A total of 110 brackets were cleaned sonically with alcohol for 15 min. For PDA functionalization of orthodontic brackets, a solution of 2 mg of dopamine hydrochloride in 10 mM tris (hydroxymethyl) amino methane buffer at pH 8.5 was prepared at specific ratios (2 mg/ml). The brackets were immersed in the prepared solution in a Petri dish and the shaking-assisted method was used for 24 h at 37 °C in darkness. Later specimens were washed with distilled water and dried and the coating process was repeated exactly with fresh solution for another 24 h to apply the second layer [22].

The control samples (Br-0) and coated samples (Br-PDA) were assessed immediately after coating and after two months of immersion in artificial saliva (aged group, see recipe below) to test the antimicrobial activity. The aging period was 14 days in artificial saliva (AS) and distilled water for coating stability while simulated body fluid (SBF) was used as an immersion solution for 14 days to test bioactivity. Throughout the experiment, the immersion solution was replaced every three days to maintain its integrity.

### Biofilm formation on freshly coated and aged coated brackets

The assessment of biofilm formation was conducted against *S. mutans*. Initially, *S. mutans* was cultured from glycerol stock in liquid Brain heart infusion (BHI) medium for 24 h at 37 °C with 5% CO_2_. Subsequently, *S. mutans* was plated onto agar plates and allowed to grow for 48 h. Individual colonies were then resuspended and cultured in BHIS medium (BHI containing 1% sucrose) for an additional 24 h under the same incubation conditions. Normalization of *S. mutans* was achieved to an optical density (OD600) of 0.5. The experiment comprised the following groups: control samples (Br), coated brackets (Br-PDA), and aged coated brackets (AG), which were immersed in a suspension of *S. mutans* (10^8^ CFU/mL, in BHIS) within 12-well plates.

Incubation was carried out for 48 h at 37 °C with 5% CO_2_. Additional samples were prepared for SEM analysis. Following incubation, all brackets were aseptically removed from the wells and transferred into 2 ml microcentrifuge tubes containing 1 ml of PBS. For colony count assessment triplicates of brackets underwent washing with sterile phosphate-buffered saline (PBS) three times. Subsequently, an additional 1 ml of PBS was added and gently shaken. Samples were then subjected to serial dilution and plating on BHI agar for quantifying planktonic/non-adherent bacteria. Following vortexing for 10 s, samples underwent ultrasonic bath sonification (Branson 1210 Ultrasonic Cleaner; Emerson; St. Louis, Missouri, USA) for five minutes to separate the biofilm and bacteria attached to the brackets [24, 25].

Serial dilution was carried out, and 100 µl of each sample was plated on BHI agar. After incubation for 48 h, bacterial colonies were counted, and colony-forming units were calculated. In a separate set of triplicates, brackets were washed with PBS three times and subsequently stained with a 0.5% (m/v) crystal violet water solution for 15 min. Following staining, the biofilms were washed with distilled water to remove excess crystal violet, and photographs of the stained biofilms were captured. Furthermore, the crystal violet within the biofilms was dissolved in 95% ethanol, and the optical density (OD) value of the crystal violet solution was recorded at 590 nm [24, 25].

### Characterization of surface biointeraction with SBF

Specimens, including coated and uncoated brackets, underwent initial washing with deionized water to eliminate contaminants before being submerged in a simulated body fluid (SBF) solution for 14 days, mimicking human blood plasma composition to assess the biointeraction of the coatings. The SBF was prepared according to Kakouba et al. [26] protocol with the concentration of an ion similar to human blood plasma, containing vital ions and anions such as Na^+^ (142 mM), K^+^ (5 mM), Ca^2+^ (2.5 mM), Mg^2+^ (1.5 mM), Cl^−^ (147.8 mM), HCO^3−^ (4.2), SO_4_^2−^ (0.5 mM), HPO_4_^2−^ (1 mM), and tris(hydroxymethyl)aminomethane as a buffering agent.

The required volume of SBF (Vs) was determined based on the apparent surface area of each specimen (Sa) to ensure proper immersion and exposure [26]. Throughout the experiment, the SBF solution was replaced every three days to maintain its integrity. Following the immersion period, the specimens were extracted from the SBF solution, and rinsed with deionized water to eliminate any residual solution or salts. Following that, the samples were dried at 60 °C in an oven to eliminate any residual moisture and were subsequently examined using a scanning electron microscope (SEM, Philips XL 30, Philips, Eindhoven, the Netherlands) [26].

### Coating stability assessment

To assess coating stability, new coated and uncoated samples were submerged in deionized water and artificial saliva for 14 days [27]. For artificial saliva (AS), the composition of saliva comprises predominantly water, constituting up to 99.5% of its content. Inorganic compounds make up a minor yet vital portion, ranging from 0.2 to 0.9%. Saliva contains various electrolytes including potassium (16.4 mM), sodium (29.3 mM), calcium (1.5 mM), magnesium (0.2 mM), and ammonium cations, which play essential roles in maintaining osmotic balance and facilitating various physiological processes [28].

The anionic group consists of phosphates (3.6 mM), carbonates (13.9 mM), chlorides (22.6 mM), rhodium, and several micronutrients. These inorganic components collectively contribute to the pH regulation, buffering capacity, and overall homeostasis within the oral cavity, highlighting their significance in oral health maintenance [28]. The surface morphology of both coated and uncoated brackets in the different solutions (artificial saliva (pH 4.5) and water for stability) was examined using a scanning electron microscope (SEM, Philips XL 30, Philips, Eindhoven, the Netherlands) [29–31].

### Measurement of pH change in water, and in SBF solution

pH is vital in the formation of hydroxyapatite in the surrounding environment thus the pH changes of immersion solutions (SBF and distilled water) were measured using a pH meter (Mettler Toledo GmbH, Erftstadt, Germany) at different time intervals. In the test, coated and uncoated samples were immersed in 5 ml of SBF and water. The pH was measured at different intervals (1 h, 24 h, 36 h, 4, and 7 days). The original SBF (pH = 7.4) was used as the control.Three samples were measured for each group [32, 33].

### Statistical analysis

Numerical variables were presented as mean values, where parametric distribution was observed, along with their respective standard deviations (SD). The statistical analysis employed in this study was a t-test: Two-Sample Assuming Equal Variances. The analysis includes t-statistic, degrees of freedom (df), and critical values to assess the significance of the observed differences between the aged, control, and coated samples.

## Results

### Antimicrobial activity against *S. mutans*

#### Biofilm formation on control, coated, and aged brackets

**Fig. 1:**
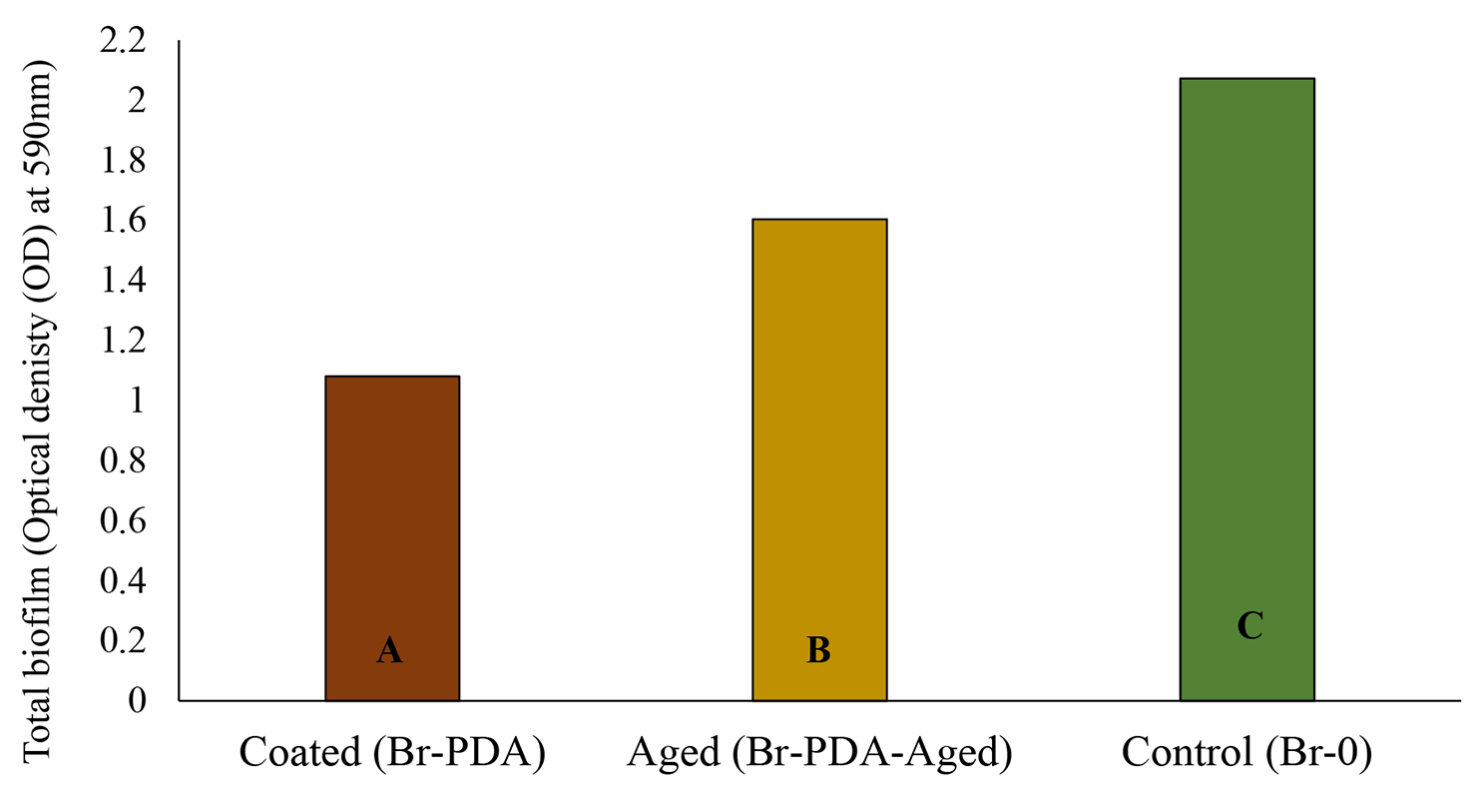
Optical Density (OD) measurements of biofilm for coated brackets (Br-PDA), 2 months-aged samples (Br- PDA-Aged), and control brackets (uncoated, Br-0). Groups that do not share a letter are significantly different

The biofilm test results indicate a significant difference in biofilm formation among the tested samples, p-value < 0.05 (Fig. 1). The control group exhibited the highest mean biofilm formation with a value of 2.072, followed by the samples aged in AS for 2 months with a mean of 1.603. In contrast, the coated samples demonstrated the lowest mean biofilm formation with a value of 1.08. Statistical analysis using a t-test assuming equal variances revealed a substantial discrepancy between the coated samples and the control group, suggesting a significant difference, p-value < 0.05. Similarly, the comparison between the samples aged in water for 2 months and the control group showed a significant difference. These results imply that the coating significantly reduced biofilm formation compared to the control group.

#### CFU (colony forming units) of *S. mutans*

**Fig. 2:**
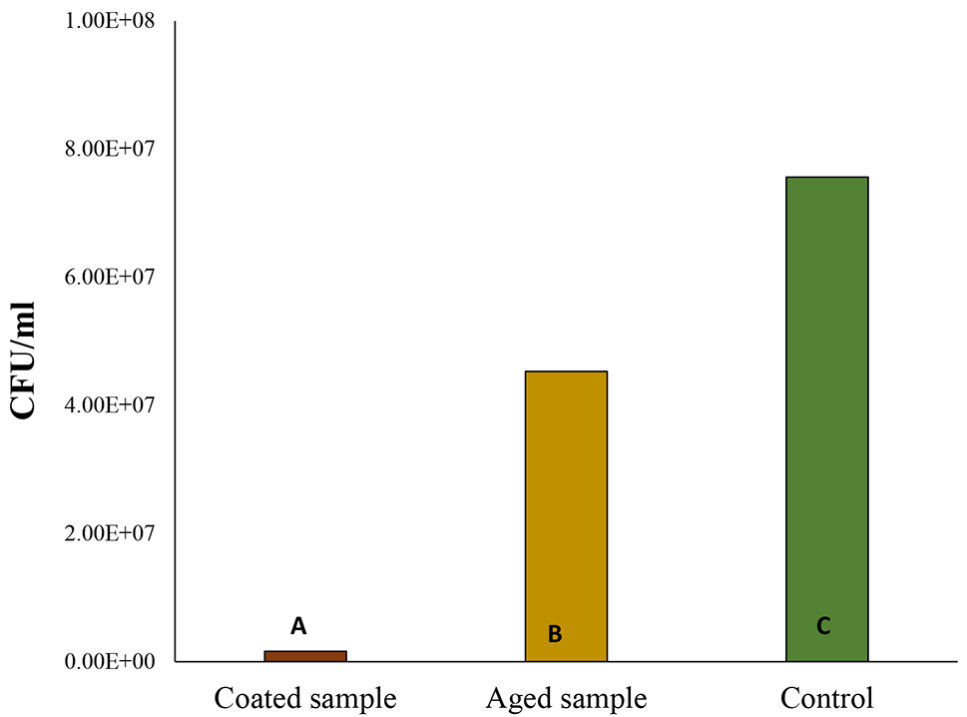
Colony forming unit (CFU) measurements of *S. mutans* biofilm growth on coated brackets (Br-PDA), 2 months-aged samples (aged Br-PDA), and control brackets (without coating). Groups that do not share a letter are significantly different

The CFU results indicate a significant difference among the coated, aged, and control samples (Fig. 2), p-value < 0.05. The coated sample exhibited significantly the lowest CFU count at 1.63E + 06, suggesting a potentially effective protective mechanism against microbial growth. The aged sample had a much higher count of 4.53E + 07 compared to the fresh-coated brackets, suggesting reduced resistance to microbial colonization over time. However, this count was still significantly lower than the control sample, which had the highest CFU count at 7.56E + 07. This indicates that the control sample lacked any protective treatment and, therefore, had the highest susceptibility to microbial proliferation.

### Characterization and surface bioactivity using SEM

**Fig. 3:**
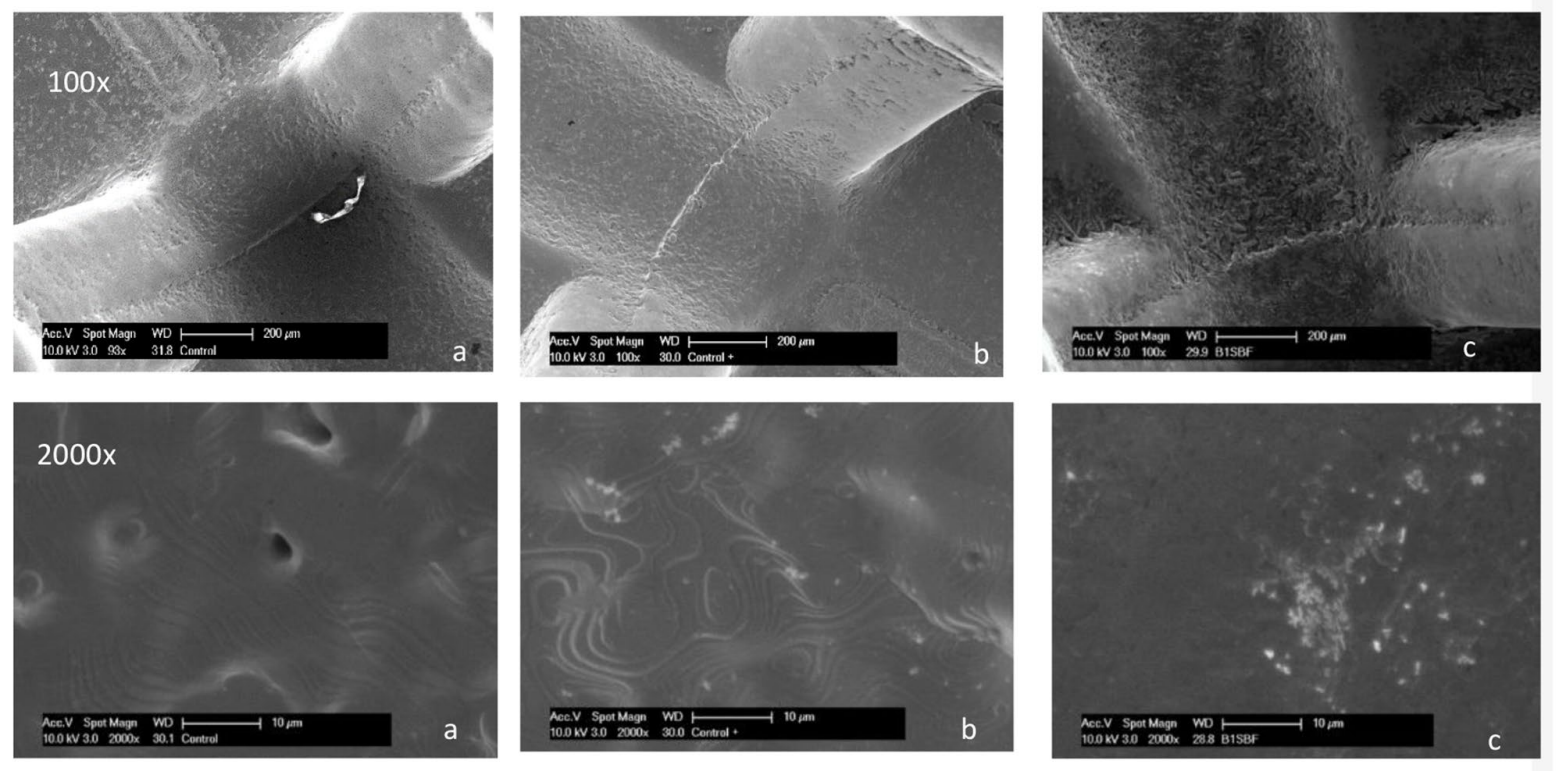
SEM micrographs at different magnifications. **a** Control brackets Br-0. **b** Coated Br-PDA before immersion. **c** Coated Br-PDA in SBF after 14 days of immersion

The bioactivity of polydopamine-coated brackets in simulated body fluid (SBF) compared to water and control conditions demonstrates a significant enhancement in the formation of apatite crystals on the coated surfaces after 14 days (Fig. 3).

### Coating stability

**Fig. 4:**
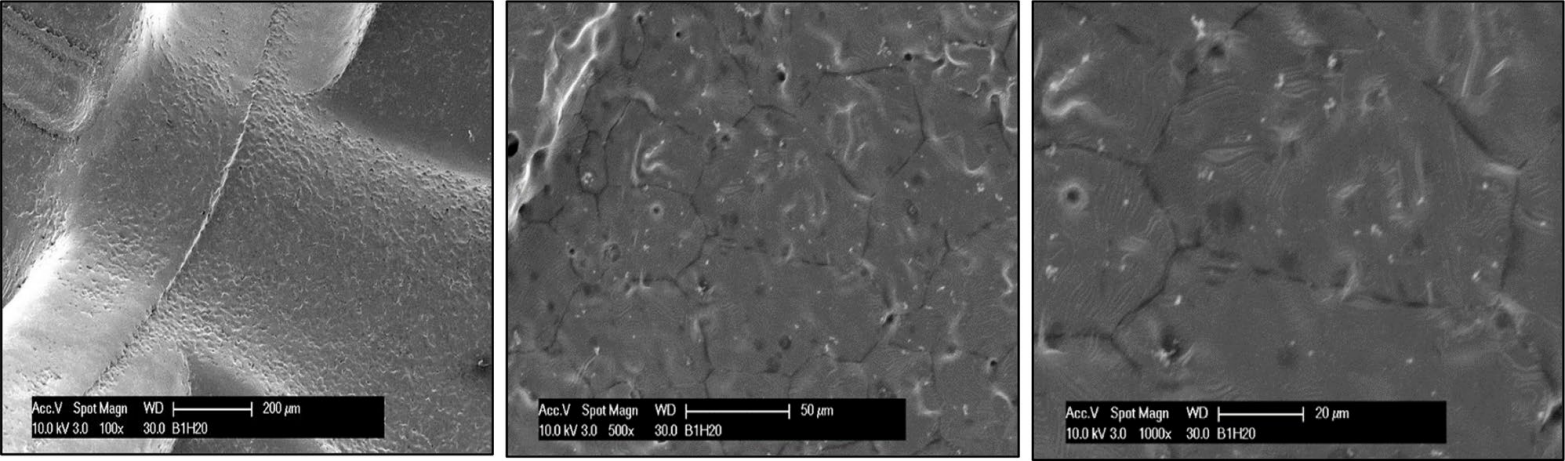
SEM micrographs of coated bracket (Br-PDA) at three different magnifications after immersion in distilled water for 14 days

**Fig. 5:**
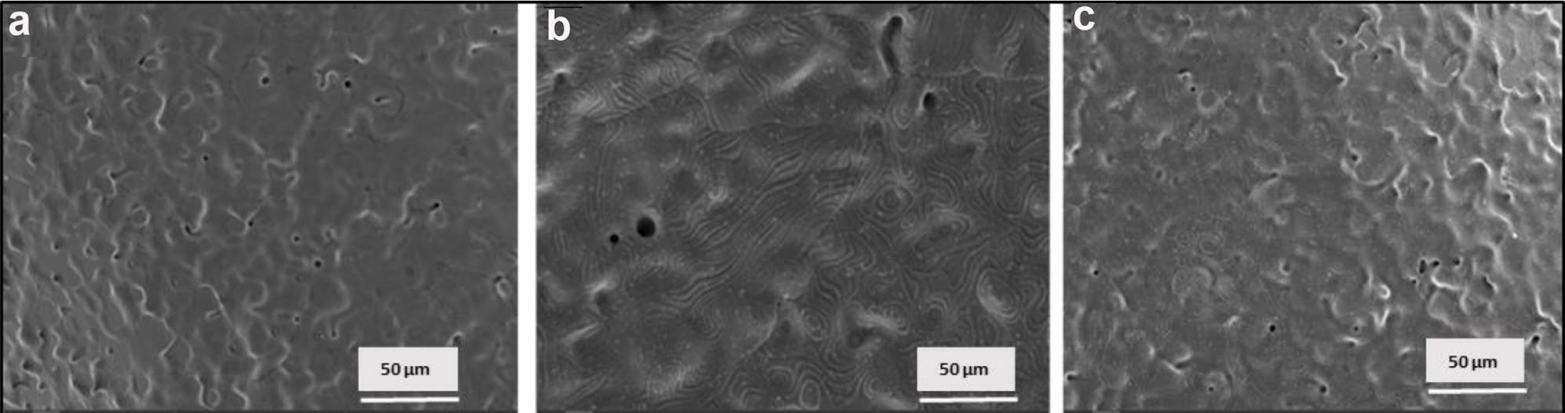
SEM micrographs of brackets (**a**) control brackets (**b**) coated bracket, (**c**) coated after immersion in AS for 2 months

The evaluation of coating stability on brackets immersed in water and artificial saliva for 14 days yielded promising results (Figs. 4 and 5). The coatings appeared homogeneous and continuous before immersion, with slight swelling observed after 14 days of immersion. While the coated brackets exhibited overall stability in both environments, a notable difference was observed in the occurrence of minute cracks. In water, the coating showed some resilience but with a few minor cracks emerging after the immersion period, whereas in artificial saliva, no cracks were observed. This suggests a higher level of durability and compatibility of the coating in conditions mimicking oral fluids.

### Measurement of pH change in water and in SBF solution

**Fig. 6:**
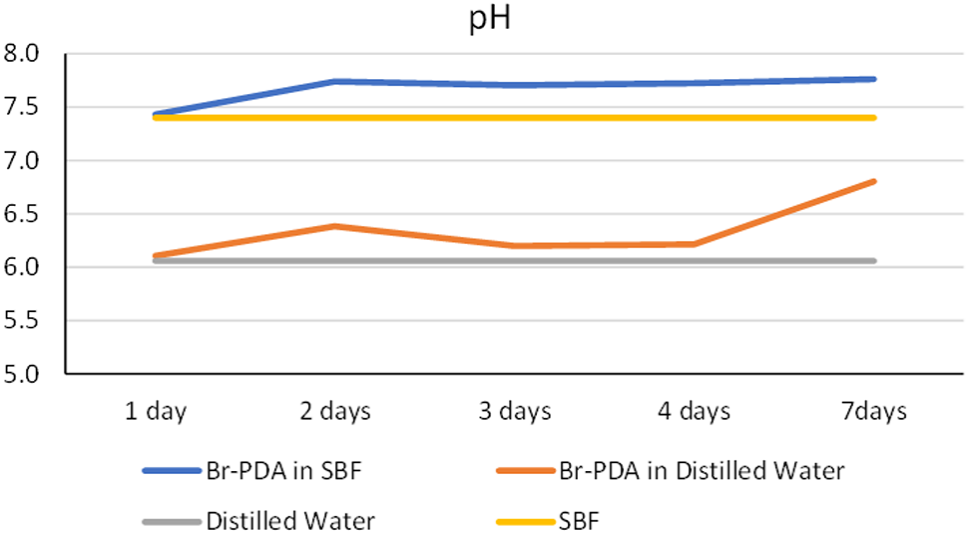
pH changes of solution throughout 7 days of coated brackets in SBF and distilled water

The results show a significant difference in the pH levels of brackets immersed in simulated body fluid (SBF) compared to those immersed in water (H_2_O) over various time intervals, p-value of ˂0.05 (Fig. 6). In the SBF solution, the pH of the brackets initially at 7.43 slightly increased after 1 h to 7.74 and remained relatively stable around 7.8 throughout the immersion period. This trend suggests that the brackets in SBF exhibited a mild alkaline buffering effect, with the pH gradually rising and maintaining a more neutral to slightly alkaline environment. In addition, the brackets immersed in water started at a lower pH of 6.108 and showed a slight increase to 6.4 after 24 h. However, the pH was raised to 6.8 after 7 days.

## Discussion

Enamel demineralization and periodontal problems are common issues faced by orthodontic patients, affecting as many as 50% of them [34]. Notably, white spot lesions (WSLs) can occur around orthodontic brackets within a month of their placement, contrasting with the usual timeframe of at least six months required for the development of typical caries [8]. These lesions frequently occur on the buccal surfaces of teeth adjacent to the brackets, with a notable prevalence in the gingival region [2].

Managing white spot lesions (WSLs) requires a comprehensive approach, with a key emphasis on preventing demineralization and inhibiting biofilm formation [35]. Additionally, implementing bioactive coatings with bacteriostatic and bactericidal properties shows promise in reducing bacterial adhesion to biomaterials, thus potentially enhancing treatment efficacy [35]. Moreover, utilizing methodologies for lesion remineralization is essential in the management of WSLs [36]. To prevent white spot lesions (WSLs), common methods include good oral hygiene with fluoridated toothpaste and mouthwash, applying varnishes, using antibacterial resins, and modifying orthodontic elastics [37–39]. More recent techniques involve adding nanoparticles to adhesives, cement, elastics, and coating orthodontic brackets, wires, and bands [40, 41].

In the current study, orthodontic brackets were double-coated with thin adherent polydopamine layers using the agitation-assisted technique (300 rpm). Compared to the the other strategies for the prevention of WSL, polydopamine (PDA)-coated brackets offer a unique advantage. Unlike traditional methods, PDA not only provides antimicrobial properties but also promotes remineralization by depositing calcium and phosphate at the bracket-tooth interface. This dual function gives PDA-coated brackets an edge, as they not only inhibit biofilm formation like other coatings but also actively contribute to preventing demineralization, offering a more comprehensive approach to WSL prevention.

The formation mechanism of PDA involves multiple complex steps, which are still a topic of discussion [42]. It has been hypothesized that under alkaline conditions, dopamine molecule undergoes oxidation to dopamine quinone, followed by intramolecular cyclization into leucodopaminechrome. Subsequent oxidation and rearrangement lead to the formation of 5,6-dihydroxy indole and 5,6-indolequinone [43]. These products, including catechol, quinone, and indole, serve as the primary constituents of the PDA structure (20). Moreover, self-polymerization of polydopamine was performed under specific shaking conditions instead of the classic static method in an alkaline solution, to obtain rough polydopamine (PDA) coatings. In a former publication, these rough PDA coatings were proven to significantly improve the antibacterial properties against different microbial strains [22].

Antimicrobial activity was tested against *S. mutans* which is the main causative organism for dental caries using biofilm density and colony-forming unit analysis [44]. The results of the study provide strong evidence for the efficacy of polydopamine (PDA)-coated orthodontic brackets in significantly reducing biofilm formation against *Streptococcus mutans*. PDA-coated samples showed a marked decrease in biofilm accumulation (1.08 OD) compared to control brackets (2.072 OD), p-value: 3.43101E-07. Even after two months of aging, the coated brackets continued to demonstrate lower biofilm formation (1.603 OD) than uncoated controls, highlighting the coating’s sustained biofilm-inhibiting properties over time. Additionally, colony-forming unit (CFU) analysis supported the protective effects of PDA coating, with the lowest CFU count (1.63E + 06) observed in coated samples. Although slightly higher CFU counts were recorded after aging, the coating still significantly reduced microbial colonization compared to controls, even after 2 months.

Polydopamine (PDA), known for its antimicrobial properties, prevents bacterial attachment and growth on surfaces by creating a barrier and complexing bacterial nutrient supplies [20]. This antimicrobial activity is attributed to redox cycling of the benzene ring of dopamine which releases ROS and exerts toxic effects on active groups on the bacteria’s protein, DNA, and lipids. Redox cycling means/comprises, that PDA can accept or donate electrons repeatedly, resulting in beneficial radical-scavenging effects and the generation of reactive oxygen species (ROS), contributing to its antimicrobial properties [13, 45]. This chemistry involves two electron transfers between quinone and hydroquinone structures. The catechol moieties of PDA donate electrons to oxygen molecules, leading to the production of hydrogen peroxide, which in turn generates hydroxyl radicals, thereby promoting localized and immediate antibacterial activity [13].

Ensuring good stability of coated intraoral appliances is crucial for the safety of patients. For polymer coatings, three primary processes typically occur during aqueous immersion: swelling, dissolution, and hydrolysis [46]. Static stability tests of polydopamine (PDA) coatings were conducted at 37 °C for 14 days in deionized water (pH = 7) and AS (pH = 4.5) to evaluate how the coating withstands both acidic and neutral environments over time. The use of a more acidic medium like AS at pH 4.5 replicates the conditions that occur during plaque formation and acid attacks in the oral cavity, providing insight into how well the coating performs in challenging environments. Distilled water was used as a neutral control to compare stability across different conditions. The 14-day period is sufficient to observe potential structural changes or degradation in the coating. The coatings appeared homogeneous and continuous before immersion, with slight swelling observed after the immersion period indicating the good stability of the PDA coatings. Such findings were further supported by the significant observed antimicrobial activity in comparison to the control after a two-month immersion period.

The pH changes were tested over 7 days in simulated body fluid (SBF) and distilled water to evaluate the polydopamine (PDA) coating’s buffering capacity. This timeframe evaluated the coating’s ability to maintain a stable environment that supports remineralization in SBF, while distilled water served as a control for neutral conditions. The rising pH observed in the brackets immersed in SBF and water is of considerable importance in dental care. It indicates the ability of the brackets or their surrounding environment to buffer acidic conditions, which is crucial for preventing dental caries [47]. Acidic conditions can demineralize tooth enamel and lead to cavities [47]. By maintaining a more neutral or slightly alkaline pH, the brackets may help buffer the acidic medium, thus reducing the risk of enamel demineralization and promoting the deposition of apatite, which is essential for tooth remineralization and preventing caries formation [48].

This hypothesis was supported by the SEM analysis, revealing the presence of apatite crystals on the bracket surface after 14 days of immersion in SBF. This could be attributed to the ability of PDA to bind with Ca^2+^ ions resulting in the nucleation of CaP crystals [49]. The deposition of apatite crystals on orthodontic brackets attached to the enamel surface of teeth may offer several benefits, particularly in enhancing the durability and health of the tooth enamel. Apatite crystals act as a natural remineralization agent, replenishing minerals lost from the enamel due to acid attacks from bacteria in the oral cavity [50]. By forming a protective layer of apatite, the enamel surface becomes more resistant to demineralization, reducing the risk of dental caries and enamel erosion during orthodontic treatment [51].

Moreover, apatite deposition serves as a buffer against acidic environments, such as those created by the fermentation of dietary sugars by oral bacteria [52]. This buffering capacity helps maintain a balanced pH in the oral cavity, mitigating the harmful effects of acid on tooth structure and promoting overall oral health [53]. Therefore, the deposition of apatite crystals on orthodontic brackets could not only strengthen the enamel but also contribute to the prevention of dental decay and erosion, supporting the long-term success of orthodontic treatment while preserving the integrity of the natural tooth structure.

One limitation of this study is its in vitro design, which may not fully simulate the complexity of the clinical conditions in the oral environment. Although simulated body fluid and artificial saliva were used, the dynamic interactions inside the oral cavity could affect the performance of polydopamine (PDA)-coated brackets. Additionally, the relatively short two-month aging period may not reflect the long-term stability of the coating during the typical duration of orthodontic treatment, which can last several years. A longer-term evaluation is needed to ensure sustained efficacy. Lastly, the study focused on a specific set of bacteria for biofilm formation. Since the oral cavity hosts a wide variety of microbial species, further studies are required to assess the coating’s effectiveness in preventing biofilm formation in a more diverse microbial environment.

## Conclusions

Based on the current findings, coating orthodontic brackets with polydopamine layers demonstrates significant antibacterial properties against *Streptococcus mutans*, the primary causative organism of dental caries. This antimicrobial efficacy originates from polydopamine’s ability to impede bacterial attachment and growth, reinforced by its redox-dependent properties that generate reactive oxygen species (ROS). Moreover, the observed increase in pH in brackets immersed in Simulated Body Fluid (SBF) emphasizes the importance of alkaline buffering in preventing caries, promoting apatite to protect against conditions like white spot lesions (WSLs). Moreover, by facilitating the deposition of apatite crystals on bracket surfaces, polydopamine may strengthen enamel, improve resistance to demineralization, and help regulate oral pH. This contributes to the success of orthodontic treatment and promotes long-term oral health. Furthermore, polydopamine’s cost-effectiveness, combined with its ease of application, makes it an attractive and sustainable option for enhancing the performance and longevity of orthodontic brackets. Future studies will extend the aging period to evaluate the long-term stability and effectiveness of the PDA coating. Additionally, the interaction with multiple bacterial species will be examined for a better understanding of the antimicrobial properties in more complex biofilm environments. Optimization of the PDA coating formulation will also be investigated to enhance performance and durability over extended use. Testing the biocompatibility of polydopamine and its impact on bonding strength in orthodontic brackets will also be essential to ensure safety and efficacy.

## Data Availability

All data generated or analyzed during this study are included in this published article.
